# Food Chemistry and Bioactive Compounds in Relation to Health

**DOI:** 10.3390/molecules30193977

**Published:** 2025-10-04

**Authors:** Leontina Grigore-Gurgu, Elena Enachi, Iuliana Aprodu

**Affiliations:** 1 Faculty of Food Science and Engineering, Dunarea de Jos University of Galati, 111 Domneasca Street, 800201 Galati, Romania; 2 Faculty of Medicine and Pharmacy, Dunarea de Jos University of Galati, 47 Domneasca Street, 800008 Galati, Romania

Bioactive compounds present in food are essential factors in maintaining optimal health, through their ability to modulate physiological processes, reduce oxidative stress, and contribute to the prevention of chronic diseases related to aging. This Special Issue of *Molecules*, entitled “Food Chemistry and Bioactive Compounds in Relation to Health”, includes ten original research articles and one review paper, manuscripts that address several advanced methods for extraction, purification, and structural characterization of bioactive compounds from food and natural sources, as well as the functional evaluation of their biological activities.

The studies included in this Special Issue can be grouped into six categories: (i) the antioxidant and anti-inflammatory potential of polyphenols, illustrated by the inhibition activity of metalloproteinases, the evaluation of the antioxidant properties of *Echinacea purpurea*, and the newest insights into the phenolic compounds from *Prunus spinosa*; (ii) the protective mechanisms against metabolic stress and cellular apoptosis induced by dicarbonyls; (iii) bioactive peptides and enzymatic hydrolysates with specific activity, including peptides that stimulate alcohol dehydrogenase activity and functional modulators obtained from brewer’s yeast; (iv) antimicrobial activities and efflux pump inhibition by sesquiterpenes (nerolidol, farnesol, α-bisabolol) formulated in nano-liposomes; (v) compositional and sensory analyses of cold-pressed oils and functional plant extracts, with risk assessment of polycyclic aromatic hydrocarbons in Chinese medicinal plants and characterization of cold-pressed plant oils; and (vi) metabolomic and prebiotic evaluations of fruit-derived compounds through the bioactive potential of micronized raspberry pomace powders and the bioaccessibility and prebiotic potential of polyphenols from black elderberry (*Sambucus nigra*). In order to motivate readers to discover the papers of this Special Issue, a short and comprehensive description is provided in the following paragraphs.

Latronico et al. [Contribution 1] investigated the effects of four dietary antioxidants, green tea extract (GTE), resveratrol (RSV), curcumin (CRC), and olive fruit extract (OLI), on oxidative stress and the activity of MMP-2 and MMP-9 metalloproteinases. The targeted enzymes are known to be involved in breast cancer progression. All tested compounds and extracts demonstrated in vitro antioxidant activity, the most effective being the GTE. On the THP-1 macrophages activated with LPS (lipopolysaccharide), GTE, RSV, and CRC significantly reduced reactive oxygen species (ROS) production and the levels of MMP-2/MMP-9, while OLI showed minimal effects. In-gel activity analysis of patient sera confirmed a dose-dependent inhibition, with GTE being the most potent overall inhibitor, and CRC and OLI exhibiting selective effects on the MMP-2 gelatinase. The results suggested that polyphenols, through several different mechanisms including oxidative stress reduction and metal ion chelation, may represent promising agents for the prevention and adjuvant therapy of breast cancer. However, to maximize the in vivo efficacy, strategies to increase bioavailability, such as the use of nanomaterial-based delivery systems, are still needed. These results were consistent with previous reports showing that the green tea catechins inhibit MMP activity, both through direct binding and modulation of ROS [1,2], and RSV reduces MMP-9 expression through the NF-κB and AP-1 pathways [1,2].

Liccardo et al. [Contribution 2] investigated the effect of genistein, an isoflavone from soy, on methylglyoxal (MG)-induced cytotoxicity in endothelial cells, a process associated with vascular complications of diabetes. The methylglyoxal compound has been identified as a promoter of oxidative stress and apoptosis, through the generation of reactive oxygen species (ROS) and activation of the mitogen-activated protein kinases (MAPKs), extracellular signal-regulated kinase (ERK/p38), and caspase-3 pathways. The results showed that genistein significantly reduced ROS production, inhibited nuclear erythroid 2-related factor (Nrf2) translocation, and blocked MAPK and caspase-3 activation, thereby preventing apoptosis. These findings suggest that genistein could be used as a therapeutic supplement to prevent endothelial dysfunction and vascular complications associated with diabetes. The obtained results are similar to those reported by Pang et al. [3], who showed that polydatin protects human endothelial cells against MG-induced apoptosis by reducing oxidative stress and maintaining mitochondrial potential [3]. Do et al. [4] also showed that genistein-rich plant extracts reduce ROS formation and apoptosis under glucotoxicity conditions.

Wang et al. [Contribution 3] investigated several corn gluten-derived peptides with alcohol dehydrogenase (ADH) activation potential, after simulated gastrointestinal digestion and absorption through an in vitro model of the human intestine with Caco-2 cell monolayers. While the hydrolysate obtained with alcalase showed the highest ADH activation activity (≈55%), the peptides fraction with a molecular weight lower than 1 kDa was the most active. Three peptides, namely SSNCQPF, TGCPVLQ, and QPQQPW, were identified in this fraction. The identified peptides exhibited potent in vitro ADH activation activity, with EC_50_ values of 1.35 mM, 2.26 mM, and 2.73 mM, respectively. Molecular docking analyses showed that these peptides bind with the active site of ADH through hydrogen bonds and hydrophobic interactions and are able to create a stable complex. Compared to chickpea peptides (EC_50_ > 3 mM) [5], those from corn showed stronger activity. When compared to the tripeptide KPC from chicken breast (binding energy −6.6 kcal/mol) [6], the SSNCQPF and TGCPVLQ peptides had lower binding energies (−8.69 and −8.20 kcal/mol), indicating a more stable interaction with ADH.

Cao et al. [Contribution 4] assayed the concentrations, sources, and health risks associated with polycyclic aromatic hydrocarbons (PAHs) in seven Chinese herbal medicines using a rapid extraction and purification method, followed by gas chromatography–mass spectrometry analysis (GC-MS). The total PAH content ranged from approximately 177 to 1414 µg/kg, with phenanthrene being the main compound. The characteristic ratios analysis indicated that the main sources of contamination were from oil, coal, and biomass combustion. Through the incremental lifetime cancer risk assessment, it was shown that honeysuckle ingestion posed a potential risk, while the *Lycium chinense* fruit posed a much lower risk. The other five investigated plants, namely ginseng, glycyrrhizae, *Coix lacryma*, and seeds of lotus and *Sterculia lychnophora*, were found to be within acceptable limits. The sensitivity analysis, carried out using Monte Carlo simulations, highlighted that PAH concentration is considered to be the main risk determinant. The study confirmed the observations of Ishizaki et al. [7] regarding the predominance of 2–3-ring PAHs in medicinal plants, suggesting the recent contamination and increased mobility throughout the environment. Also, the level of benzo[a]pyrene (BaP) detected in the honeysuckle samples exceeded the safety limit set by European regulations, which is very similar to the results obtained by Yu et al. [8] in the analysis of PAHs in medicinal plants used as food additives.

Adebimpe Ojo et al. [Contribution 5] evaluated the antioxidant properties of flowers and leafy stems of *Echinacea purpurea* grown with synthetic mulches of different colors (black, green, brown) and thicknesses (80 and 100 g/m^2^). The panel of analyses included chemical composition (protein, fat, ash), total polyphenol content, and antioxidant activity (DPPH, ABTS, FRAP), as well as polyphenol profile by HPLC. The results showed that the flowers had significantly higher protein, ash, and polyphenol contents and antioxidant activity compared to the stems and leaves. The main phenolic compounds identified were p-coumaric acid, chlorogenic acid, and rutin. The mulch did not significantly influence the overall chemical composition but instead caused variations in the concentration of some polyphenols, thus confirming the potential of *E. purpurea* flowers as a source of natural antioxidants for functional food and cosmetic and pharmaceutical products. The total polyphenol concentrations in the flowers were within the range reported by the study of Tsai et al. [9], hence confirming the high antioxidant potential of this species. Moreover, rutin was identified as the major flavonoid in all the flower samples, complying with the observations of Skrzypczak-Pietraszek et al. [10], who highlighted the essential role it plays in the antioxidant activity of plant extracts.

Santana et al. [Contribution 6] compared the antibacterial activity and efflux pump (NorA, Tet(K), MsrA, MepA) inhibition capacity of the nerolidol, farnesol, and α-bisabolol sesquiterpenes, used individually and as liposomal nanoformulations, against multidrug-resistant strains of *Staphylococcus aureus*. In vitro tests showed that the isolated sesquiterpenes exhibited direct antibacterial activity and reduced the minimum inhibitory concentrations (MIC) of the antibiotics and ethidium bromide (EtBr), indicating the inhibition of efflux pumps. Nerolidol reduced the MIC of EtBr to 5 µg/mL for the 1199B strain, exceeding the standard carbonyl cyanide 3-chlorophenyl hydrazone inhibitor, and farnesol exhibited the lowest MIC (16 µg/mL) against the strain RN4220. Liposomal nanoformulations did not significantly improve the activity, except for farnesol liposomes, which reduced the MIC of EtBr to 40.3 µg/mL. The study results align with the observations of de Moura et al. [11], who demonstrated the efficacy of nerolidol against multidrug-resistant bacteria, including by inhibiting the formation of biofilm. Also, da Cruz et al. [12] highlighted the ability of α-bisabolol to inhibit Tet(K) and NorA pumps, and Oliveira et al. [13] showed that farnesol can potentiate the effect of conventional antibiotics, such as fusidic acid, against *S. aureus* strains expressing the MrsA mechanism.

Rabiej-Kozioł et al. [Contribution 7] presented a comprehensive characterization of the chemical composition, antioxidant activity, oxidative stability, and sensory attributes of six cold-pressed oils, namely flax (*Linum usitatissimum*), pumpkin (*Cucurbita pepo*), milk thistle (*Silybum marianum*), rapeseed (*Brassica napus*), camelina (*Camelina sativa*), and sunflower (*Helianthus annuus*), commercialized on the Polish market. The analyses included the determination of the fatty acid profile, the quantification of the content of tocopherols (44.04–76.98 mg/100 g), sterols (300–684 mg/100 g). and polyphenols (2.93–8.32 mg GA/100 g), as well as the evaluation of antioxidant activity by DPPH, ABTS, and FRAP methods, with values ranging between 185.36 and 396.63 μmol TE/100 g, 958.59 and 1638.58 μmol TE/100 g, and 61.93 and 119.21 μmol TE/100 g, respectively. The calculated nutritional indices (AI, TI, HH) revealed a beneficial potential for cardiovascular health, most pronounced in the case of rapeseed oil (AI = 0.02; HH = 19.53). The oxidative stability, expressed by the induction period (IP), ranged between 4.87 h (linseed oil) and 12.93 h (rapeseed oil), being correlated with the proportion of polyunsaturated fatty acids (PUFAs) and the level of antioxidant biocompounds. All the samples respected the legal limits concerning the content of polycyclic aromatic hydrocarbons (PAH ≤ 8.76 μg/kg). The descriptive sensory analysis (QDA) and the hedonic tests indicated the highest acceptability for pumpkin oil, attributed to the sweet and roasted aromatic note, while the bitter taste and astringency decreased the scores for linseed oil. The results are similar to the studies of Grajzer et al. [14], who assessed the role of tocopherols in oxidative stability, and to those of Symoniuk et al. [15,16], who confirmed the variability in the composition and stability of cold-pressed oils, depending on the vegetable source and processing. The study also expands the results of Bou Fakhreddine and Sánchez [17], who showed that the sensory perception significantly influenced the purchase intention, even in the case of products with well-known nutritional benefits.

Różyło et al. [Contribution 8] investigated the effects of micronization on the bioactive composition and antioxidant properties of raspberry pomace powder (*Rubus idaeus* L.). Ball mill micronization reduced the average particle size from 225 μm (control) to 25 μm (after 10 min of micronization) and 10.5 μm (after 20 min of micronization), with minor changes in their color. They also performed an FTIR analysis that revealed changes in characteristic bands (~1720, 1635, and 1326 cm^−1^), indicating the breaking of the intramolecular hydrogen bonds in polysaccharides, and the increase in the proportion of simple sugars. The HPLC confirmed higher levels of glucose and fructose in the micronized samples. Nine types of phenolic compounds (including ellagic acid derivatives, anthocyanins, and rutin) were identified, and their concentration increased significantly after micronization. Also, the antioxidant activity (ABTS, FRAP) and total phenolic content (up to 23.94 mg GAE/g) were both higher in the micronized samples, while the DPPH-based method revealed slightly decreased activity. The results were in agreement with the studies of Sadowska et al. [18], who concluded that fluidized-jet micronization increased both the anthocyanin content and antioxidant activity in raspberry powder. The results were also validated by the observations of Różyło et al. [19] regarding the effects of micronization on phenolic composition and antioxidant activity of spinach leaves.

Dumitrașcu et al. [Contribution 9] determined the effect of enzymatic hydrolysis on the antioxidant activity and functionality of proteins from spent brewer’s yeast (SY). Three proteases (bromelain, neutrase, and trypsin) were used for SY hydrolysis for a period of time between 4 and 67 h, and the obtained hydrolysates were characterized from physicochemical, antioxidant, and techno-functional points of view. Neutrase generated the highest degree of hydrolysis and the highest levels of soluble proteins and antioxidant activity (ABTS^+^ and DPPH), while bromelain produced the <3 kDa fraction with the highest DPPH antioxidant activity. Trypsin led to the best foaming properties, and bromelain provided emulsions with the highest structural rigidity. The SDS-PAGE analyses confirmed the predominance of peptides with molecular weights < 10 kDa in all hydrolyzed samples. From a functional point of view, trypsin hydrolysates had the best foaming capacity and stability (up to 132.5% and 98%), and bromelain-based hydrolysate emulsions showed the highest structural rigidity. The results reported by Dumitrașcu et al. (Contribution 9 of this Special Issue) are in agreement with previous studies available in the literature. Marson et al. [20] reported a significant increase in the antioxidant activity of yeast hydrolysates obtained with Protamex™ (Novozymes A/S, Bagsværd, Denmark) and Brauzyn^®^ (Prozyn, São Paulo, Brazil), whereas Vieira et al. [21] confirmed the efficiency of neutrase in obtaining fractions with high antioxidant potential after ultrafiltration. Also, Mirzaei et al. [22] determined the antioxidant and ACE inhibitory activity of peptides with a molecular weight lower than 3 kDa, obtained from Saccharomyces cerevisiae with trypsin, hence further supporting the results of the abovementioned study regarding the bioactivity of small peptides.

Haş et al. [Contribution 10] investigated the bioactive potential of black elderberry (*Sambucus nigra* L.) from the spontaneous Romanian flora, focusing on the antioxidant and antimicrobial activity and bioaccessibility of phenolic compounds and their prebiotic potential. The methanolic extract of the freeze-dried elderberry powder displayed high antioxidant activity, with maximum FRAP values of 185 µmol Fe^2+^/g DW, and significant antimicrobial activity, especially against *Staphylococcus aureus* and *Candida parapsilosis* (MIC = 1.95 mg/mL). The HPLC-DAD-ESI-MS analysis identified 12 phenolic compounds, with anthocyanins being predominant (41.8%), especially cyanidin–glucosides and cyanidin–sambubiosides. Following the simulated digestion, a bioaccessibility of 74.54% was observed, with a remarkable increase in the hydroxybenzoic acid derivatives (224.64%) and thus explaining the transformation of anthocyanins into more stable compounds. The study demonstrated for the first time the prebiotic potential of the extract on five probiotic strains, with the highest growth index (GI = 152.44%) being observed for *Lactobacillus casei* at 1.5%. The results showed many similarities with the results of Imenšek et al. [23], scientists who reported similar values for DPPH and FRAP in *S. nigra* fruits. These authors reported significantly higher ABTS values, suggesting variations depending on the degree of ripening and the plant’s geographical origin. Furthermore, Reider et al. [24] assessed the prebiotic effect of a purified elderberry extract on microbial diversity, especially on *Akkermansia* spp. In addition, the studies of Mohammadsadeghi et al. [25] confirmed the antifungal activity of an elderberry extract on *Candida albicans*, although without any previous data on *C. parapsilosis*, which underlines the novelty of the current contribution.

Negrean et al. [Contribution 11] conducted a comprehensive analysis of the bioactive compounds from blackthorn fruits (*Prunus spinosa* L.), thus highlighting their antioxidant and antimicrobial potential and applications in the food, pharmaceutical, and cosmetic industries. The fruits contained several antioxidants such as flavonoids (quercetin, rutin, catechins), phenolic acids (chlorogenic, caffeic), anthocyanins (cyanidin-3-glucoside), tannins, vitamins (C), and minerals. These compounds exhibit antioxidant, anti-inflammatory, antidiabetic, antihypertensive, and antitumor effects. Other studies have demonstrated strong correlations between polyphenol content and antioxidant activity (DPPH, ABTS, FRAP), as well as antimicrobial effects against Gram-positive and Gram-negative bacteria. The extracts showed antidiabetic potential by inhibiting α-glucosidase and anti-inflammatory effects by stimulating IL-10 secretion. Antiproliferative effects on various tumor cell lines (MCF-7, HepG2) and protection against in vivo oxidative stress have also been reported. Modern extraction techniques allow for the yield of polyphenols to be increased, and the extracts can be used as natural colorants, preservatives, ingredients for supplements, and cosmetics with a sunscreen factor. The conclusions emphasized the need for further studies to clarify the bioactive mechanisms and develop functional products and smart packaging based on blackthorn extracts. The study of Marcetić et al. [26] confirmed the phenolic profile and prebiotic activity of blackthorn fruits, and the study of Magiera et al. [27] highlighted the anti-inflammatory effects ex vivo on human immune cells. Also, Condello et al. [28] demonstrated the effectiveness of a blackthorn extract in inhibiting the in vivo growth of colorectal tumors, supporting the oncological potential of these compounds.
